# Graphene oxide down-regulates genes of the oxidative phosphorylation complexes in a glioblastoma

**DOI:** 10.1186/s12867-018-0119-2

**Published:** 2019-01-03

**Authors:** Maciej Szmidt, Adrian Stankiewicz, Kaja Urbańska, Sławomir Jaworski, Marta Kutwin, Mateusz Wierzbicki, Marta Grodzik, Beata Burzyńska, Monika Góra, André Chwalibog, Ewa Sawosz

**Affiliations:** 1 Department of Morphological Sciences, Warsaw University of Life Sciences, 02-787 Warsaw, Poland; 2 Department of Molecular Biology, Institute of Genetics and Animal Breeding, Polish Academy of Sciences, 05-552 Jastrzebiec, Poland; 3 Department of Animal Nutrition and Biotechnology, Warsaw University of Life Sciences, 02-787 Warsaw, Poland; 4 Department of Genetics, Institute of Biochemistry and Biophysics, Polish Academy of Sciences, 02-106 Warsaw, Poland; 5 Department of Veterinary and Animal Sciences, University of Copenhagen, Groennegaardsvje 3, 1870 Frederiksberg, Denmark

**Keywords:** Graphene, Graphene oxide, Glioblastoma, Gene expression, OXPHOS

## Abstract

**Background:**

Recently different forms of nanographene were proposed as the material with high anticancer potential. However, the mechanism of the suppressive activity of the graphene on cancer development remains unclear. We examined the effect of oxygenated, reduced and pristine graphene on the gene expression in glioblastoma U87 cell line.

**Results:**

Conducting microarrays and RT-qPCR analysis we explored that graphene oxide (rather than reduced graphene oxide and pristine graphene) down-regulates the mRNA expression of mitochondrial oxidative phosphorylation (OXPHOS) nuclear genes of complexes I, III, IV and V. The presented results provide first evidence for the hypothesis that the suppressed growth of GBM can be the consequence of down-regulation of OXPHOS protein expression and decreased ATP level.

**Conclusions:**

We suggest that changes in the expression of OXPHOS genes identified in our study may mediate the anti-proliferative and anti-migratory effects of graphene oxide in glioblastoma cells. However, further investigations with different cell lines, regarding expression, regulation and activity of OXPHOS genes identified in our study is necessary to elucidate the mechanism mediating the anti-proliferative and anti-migratory effects of graphene oxide in glioblastoma cells.

**Electronic supplementary material:**

The online version of this article (10.1186/s12867-018-0119-2) contains supplementary material, which is available to authorized users.

## Background

Graphene is a nanomaterial with unique properties and great application potential. It is expected to revolutionize various medical fields, including oncology. Many biomedical applications of graphene and its derivatives have been proposed in cancer diagnosis [1], biomedicine [2], photothermal therapy [3, 4], cancer cell imaging [5], and drug delivery [6, 7]. Graphene is a single atomic layer of sp2-bonded carbon nanostructure [8], with a higher ratio of peripheral to central carbon atoms compared to similar nanomaterials. Graphene appears to be biocompatible, with preferential affinity to the cell membrane, and is less toxic than other carbon nanoparticles [9, 10]. Regarding biomedical applications, graphene oxide (GO) and reduced graphene oxide (rGO) are the most commonly described [11]. One of the crucial differences between the forms is their hydrophobic (rGO) or hydrophilic (GO) characteristic [12]. We previously performed the comparative toxicity studies, evaluating their effect on organism development and cancer growth in both in vitro and in vivo models [13–16]. Furthermore, it has been reported that nanocarbons can affect cell morphology and viability and can also influence DNA damage, RNA efflux and gene expression [1, 17, 18]. Hydrophilic GO possesses a large aromatic surface with reactive COOH and OH groups which facilitate connections with cellular molecules [19]. Compared to other graphene types, GO is smaller, possesses smooth edges and forms more regular structures. Based on the comparative studies previously published, we decided to perform the presented experiments using 100 nm graphene platelets in the concentration of 25 ppm [12]. The detailed description of graphene structure and its distribution within glioblastoma cells were previously extensively analyzed [12].

It was demonstrated that GO effectively inhibits tumor-sphere formation in breast, ovarian, prostate, lung, pancreas and brain cell lines [16, 17]. GO can suppress the development and migration of cancer cells by impairing mitochondrial respiration [20, 21]. Also, in vivo studies presented GO to be a suppressing agent of cancer development [22].

In the chicken embryo model, we have demonstrated that GO and rGO significantly decreased volume and weight of the glioblastoma tumor (GBM) [15, 23, 24]. Furthermore, it has been indicated and that functionalized GO might be applied as the drug delivery agent in the GBM therapy [25]. Moreover, after treatment with rGO, the apoptosis markers were significantly increased, suggesting that rGO may be involved in the inhibition of tumor development.

Importantly, graphene can directly and physically interact with DNA, causing the deregulation of gene expression [17]. GO treatment at concentrations of 10 and 100 mg/mL altered gene expression patterns, and mediated DNA-damage control, cell apoptosis, cell cycle, and metabolism [1].

Based on these investigations and continuing our previous research indicating the suppressive effect of GO and rGO on GBM growth, we hypothesized that different graphene forms (GO, rGO and pG—pristine graphene) may inhibit GBM development by regulation of the genes encoding proteins responsible for mitochondrial oxidative phosphorylation (OXPHOS). The presented study is considered as the first step in validating this hypothesis.

## Results

**Fig. 1:**
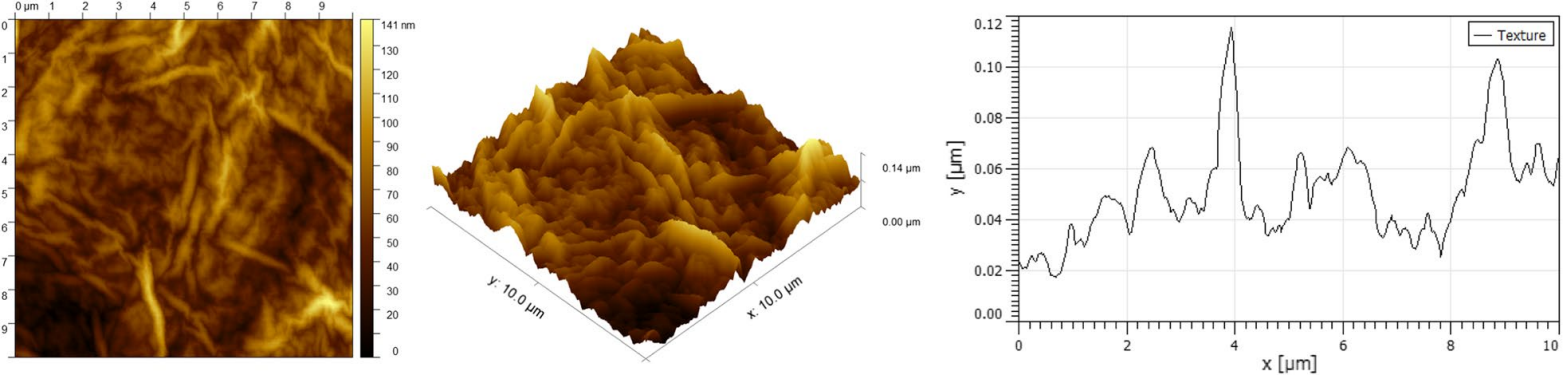
Surface morphology and line profile of graphene oxide nanoparticles. Analyzed using atomic force microscopy

Using microarrays, we have analyzed transcriptomic patterns elicited in GBM cells by treatment with either GO, rGO or pG. GO surface profile is presented on Fig. 1. GO treatment increased the expression of 90 and decreased the expression of 227 known transcripts in GBM cells. All probes reporting statistically significant differences between experimental groups are presented in Additional file 1. The treatment of GBM cells with pG changed the expression of FOS and C8orf4 genes, while GR changed only the expression of the FOS gene. Thus, we propose that rGO and pG do not notably modulate mRNA expression levels, at least under the conditions of the current experiment. Using the Enrichr tool [26] we performed enrichment analysis on the list of genes deregulated by GO treatment.

We have identified multiple overrepresented terms (Additional file 2) including: 120 biological processes, 25 cellular components, 17 molecular functions, 42 biological pathways, 291 unique transcriptional regulators (Additional file 2) and 13 unique hub proteins or protein complexes. Data from several independent databases, queried in our enrichment analysis, showed that genes involved in the activity of the electron transport chain were robustly overrepresented in our list of differentially expressed genes.

**Fig. 2:**
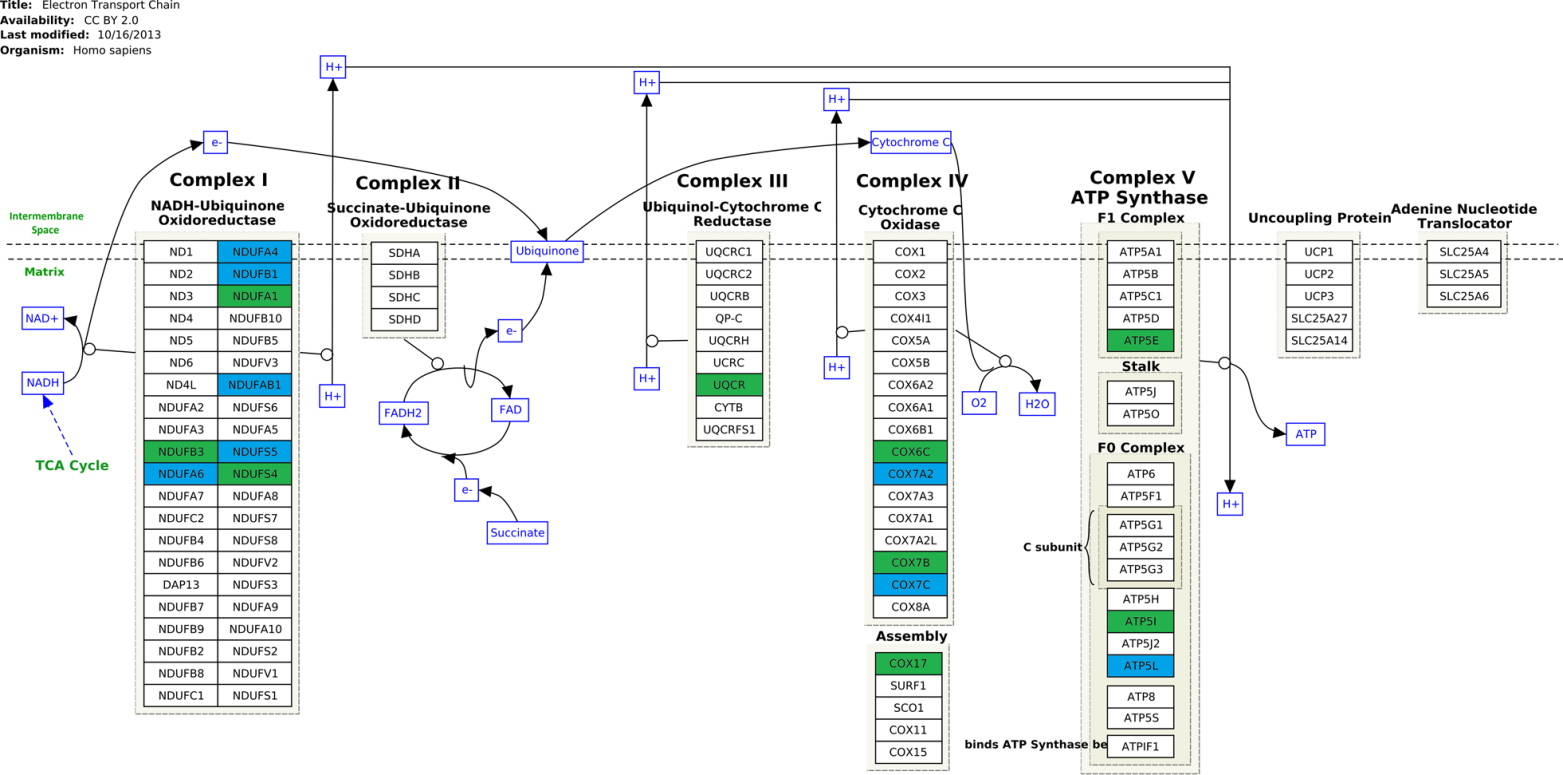
Differentially expressed genes connected to electron transport chain pathway. The scheme shows electron transport chain pathway and was downloaded from WikiPathways database. The blue, downward-pointing arrows show genes, which were down-regulated in GBM cells after GO treatment. Here, we list differentially expressed genes, that are not shown on the scheme, but that are connected to electron transport chain: ATP5EP2 (connected to overrepresented term: ATP synthesis coupled proton transport—GO:0015986), COA6 (cytochrome-c oxidase activity—GO:0004129), COX14 (Respiratory electron transport—Reactome), UQCRHL (Electron Transport Chain (Homo sapiens)—WikiPathways), UQCRQ (Electron Transport Chain (Homo sapiens)—WikiPathways), USMG5 (mitochondrial proton-transporting ATP synthase complex—GO:0005753)

Additional file 1 presents identified terms related to the electron transport chain, which were characterized in our analysis by the highest adjusted *P*-values of enrichment. Figure 2 presents the differentially expressed genes associated with the electron transport chain pathway.

**Fig. 3:**
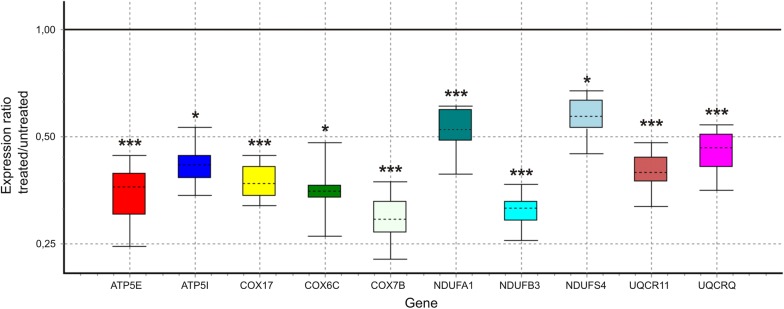
RT-qPCR analysis of selected, OXPHOS-related differentially expressed genes. Here we show relative (GO-treated vs. control cells) expression of given gene. Expression values for the given gene were first normalized to expression values of reference genes, and then compared between experimental groups. Expression ratios were calculated using the REST 2009 software tool. Results are presented as whisker-box plots where the box area encompasses 50% of all observations, the dashed line represents the sample median and the whiskers represent the outer 50% of observations. The black solid line defines the value of no change in relative expression. Statistical significance: *p < 0.05, ***p < 0.001

Microarray results were validated by RT-qPCR. We have analyzed 10 genes involved in the electron transport chain. The results of RT-qPCR analysis confirmed the findings of the microarray study (Fig. 3). The correlation coefficient between the results of RT-qPCR and microarray analyses, calculated using the Spearman rank correlation method, was equal to 0.88.

## Discussion

It was already widely presented that GO suppresses the growth of the different cancers in both in vivo and in vitro studies, however, the mechanism responsible for the inhibition remains unknown [22, 25, 27, 28].

In the present work, we used U87 cell line from the American Type Culture Collection. The studies of U87 cell markers [29] and recent genetic profiling and transcriptome analysis in human glioma cell lines indicate that this cell line is a glioblastoma cell line [30]. The comparison studies between different glioma cell lines treated with graphene were previously evaluated in our laboratory and thoroughly described [6].

Using microarrays, we showed for the first time on the U87 cell line, that GO (but not rGO and pG) down-regulates the genes responsible for OXPHOS. The differentially expressed genes detected in the presented experiments, after GO, rGO and pG treatment of GBM cells, are reported in Additional file 1 and Fig. 3. Figure 3 shows the identified genes for which the differential expression was confirmed via RT-qPCR analysis. Both experimental methods show that GO down-regulates the mRNA of OXPHOS nuclear genes of complexes I, III, IV and V.

Interestingly, our experiments did not detect any changes in the expression of the analyzed genes after rGO and pG treatment. We assume that the observed impact of GO on gene expression is the result of its unique chemo-physical properties. Compared to rGO and pG, GO is hydrophilic and possesses reactive COOH and OH groups, which facilitates its connection to DNA [1, 19, 31]. The anticancer activity of rGO and pG observed in other studies [14, 15] could be the result of different cytotoxic mechanisms. In contrast to GO, rGO and pG forms remain surrounded by the active and conductive delocalized electrons, which may inhibit cell viability via ROS generation and/or affect membrane integrity [32, 33]. Moreover, hydrophobic rGO shows lower water solubility than GO and frequently aggregates in salt-containing physiological buffers due to its different electrostatic charge [30]. rGO commonly forms nonspecific bindings to proteins and lipids. Strong hydrophobic interactions of rGO with the cell membrane lipids might result in its accumulation, which may eventually lead to cell membrane damages. Surface modifications of graphene (like addition of oxygen groups) improve its solubility in water. GO does not affect cell membranes as effectively as rGO, so it remains longer within the cells and consequently it may impact the cell molecular mechanisms [30]. Nevertheless, the cytotoxic activity mechanisms for rGO and pG need to be evaluated; however, it was not the primary interest of the presented study.

The function of electron transport chain Complex I is to remove electrons from NADH and transfer them to ubiquinone. It is one of the main sites of ROS production [34]. Among the other differentially regulated genes revealed in the microarray analysis, RT-qPCR confirmed that GO modulates expression of the following genes: NDUFA1, NDUFB3 and NDUFS4. The proteins encoded by those genes were described as critical for complex I functioning [35]. Decreased Complex I activity may reduce cancer growth and induce cell death via oxidative stress [36].

Complex III is involved in the GBM development and mediates resistance to drugs for glioma [37, 38]. Transcripts of UQCR11 and UQCRQ, the down-regulation of which was verified in our study via RT-qPCR, were also described to be regulated in carcinoma [39, 40]. Mutations of genes involved in complex III and IV activity were shown to be associated with glioblastoma growth [38]. Moreover, increased cytochrome c oxidase (Complex IV) activity was associated with the acquisition of chemoresistance in GBM [41]. Our RT-qPCR analysis confirmed that mRNAs of COX7B and COX17 genes, which protein products participate in Complex IV, were down-regulated Interestingly COX17 was proposed as the therapeutic target in lung cancer [42], while COX7B was shown to be overexpressed in carcinoma and its expression was decreased by anti-tumor agents in the glioblastoma treatment [43]. Complex V functions as an ATP synthase. ATP5E transcript that was identified as down-regulated in our experiment was proposed as the tumor marker in thyroid cancer [44].

We hypothesized that the significant changes in gene expression might notably affect the tumor development and OXPHOS activity. Our results, presenting down-regulation of OXPHOS mRNA expression correspond with the protein level changes reported by Zhou et al. [21]. The authors showed decreased protein level in the mitochondrial electron transfer chain complexes in breast cancer cells exposed to GO via quantitative proteomic analysis. Interestingly, they showed that GO treatment increased COX17 protein level, while we found down-regulation of COX17 mRNA. Unfortunately, the publication did not include the data of other proteins encoded by genes with deregulated mRNAs detected in our study. Most of the other genes presented in the abovementioned study were down-regulated. It was presented that the proteins of the subunits comprising every complex were also therefore down-regulated. Also, in another work [20], it was shown that graphene oxide inhibits the activity of electron transport complexes I, III and IV in a dose-dependent manner. Similarly, we found that GO inhibits the expression of genes encoding proteins belonging to the same OXPHOS complexes. It was concluded [20] that the activity of the electron transport chain proteins was decreased as the nanographene affected the iron sulfur centers of the complexes. Our research indicates that the activity of OXPHOS may also be decreased via the down-regulation of nuclear genes.

Deregulation of cellular energy metabolism is necessary to effectively support neoplastic proliferation (reviewed [45]). It was described that some of the cancer cells meet their energy demands mostly by glycolysis followed by lactic acid fermentation, as opposed to typical cells, which preferentially use mitochondrial oxidative phosphorylation. This phenomenon is known as a Warburg effect [46]. Nevertheless, both, the mentioned mechanism and a high ATP level are required for the growth of cancer [21]. GO significantly decreased the proliferation of MDA-MB-231, MDA-MB-436 and SK-BR-3 breast cancer cells via the down-regulation of OXPHOS activity without any changes in glycolysis [21]. Decreased OXPHOS activity inhibits the migratory and invasive characteristics of cancer [21].

Moreover, the growing GBM is composed of different subpopulations of cells and the population of cancer stem cells (CSC) seems to be crucial for tumor initiation and self-renewal [47, 48]. Independent of the glycolysis and differently from differentiated tumor cells, CSC activity fully depends on OXPHOS [48, 49]. Therefore, the inhibition of OXPHOS in GBM may suppress tumor expansion via the disruption of CSC functioning [48].

Disrupted OXPHOS significantly reduces ATP production and cytoskeletal function, which consequently affects the migratory and invasive activity of cancer [20]. Decreased energy production in cancer cells impairs their metastasis potential. Migration of the cells is the result of the lamellipodia or filopodia movement, which is regulated by the cytoskeleton [20, 50]. It was also shown that GO disrupts F-actin cytoskeletal functioning [20, 21]. In vivo experiments in mice revealed that GO inhibits the migration of metastatic cancer nodules [21]. Our results provide further support for the hypothesis that the suppressed growth of GBM can be the result of down-regulation of OXPHOS protein expression and decreased ATP levels. Such growth suppression may inhibit cancer migration and metastasis.

We also propose that down-regulation of the expression of nuclear genes encoding mitochondrial proteins may be a result of the mitochondrial dysfunction caused by GO. We previously reported that chicken embryo cells treated with different types of graphene possessed disrupted mitochondria [16] and GBM cells grown *in ovo* and treated with GO and rGO had degraded mitochondria [15]. Furthermore, mitochondrial fluorescence was quenched by graphene, suggesting that graphene nanoparticles were located inside and around the mitochondria of cancer cells [18]. The overproduction of ROS by the mitochondrial electron transport chain may be one of the mechanisms of GO mediated mitochondria disruption. It was suggested [51] that ROS can be involved in the toxic effects of graphene-based nanomaterials. The other researchers indicated that plasma membrane damage and oxidative stress are the key factors in graphene-induced cytotoxicity of HepG2 cells [52]. In murine macrophages, GO-induced cytotoxicity through depletion of the mitochondrial membrane potential, increasing the production of intracellular ROS and triggering apoptosis [53]. Interestingly, mitochondrial dysfunction and altered dynamics (processes of fusion and fission) were shown to regulate migration and invasion of the cancer cells [54]. Importantly, it was shown that dysfunctional mitochondria can regulate nuclear gene expression (reviewed [55]). Gene expression changes can be induced by iron-sulfur cluster (ISC) biogenesis, which is altered in dysfunctional mitochondria [56]. Based on previous studies and our present research, we speculate that GO affects mitochondrial function of cancer cells, not only via a previously proposed direct impact on ROS generation, but also via modulation of expression of the genes involved in mitochondrial activity.

## Conclusions

We demonstrated that GO treatment caused changes in the mRNA expression of genes involved in OXPHOS in GBM cells, while pG and rGO had no effect. We suggest that changes in the expression of OXPHOS genes identified in our study comprise an interesting candidate mechanism which explains the anti-proliferative and anti-migratory effects of GO in GBM cells. Further studies of tumor proliferation, protein expression panel and the effects of knock-out of genes identified in current study, are still necessary to further evaluate the presented hypothesis.

## Methods

### Nanoparticles

pG powder, produced by liquid-phase exfoliation of graphite, was purchased from Skyspring Nanomaterials (Huston, TX, USA). GO and rGO were obtained from the Institute of Electronic Materials Technology (Warsaw, Poland). GO was prepared by a modified Hummers method from natural graphite flakes (Asbury Carbon). The Zeta potential of pG, GO and rGO suspended in milli-Q water was measured on a Zetasizer Nano-ZS90 (Malvern Instruments, Worcestershire, UK). For details of graphene powder preparations cf. [16].

### AFM graphene oxide structure analysis

200 µl of graphene oxide suspension was placed on the silicic substrate, previously polished and cleaned in the preparation for atomic force microscope (AFM) measurement. The samples were dried in an exicator for 24 h. Afterwards the surface topography of the prepared samples was analyzed in the AFM measurement system (Nanosurf FLEX-Axiom). The scan sizes areas were analyzed in the range of 5 × 5 to 20 × 20 µm. The measurements were conducted in the tapping mode. The time of line scanning and amplitude of oscillation were matched properly for each analyzed sample.

### Cell culture and treatment

Human glioblastoma cell line U87 was purchased from the American Type Culture Collection (Manassas, VA, USA). The cells were maintained in Dulbecco’s modified Eagle’s culture medium containing 10% fetal bovine serum (Life Technologies, Houston, TX, USA), 1% penicillin and streptomycin (Life Technologies) at 37 °C in a humidified atmosphere of 5% CO2/95% air in a DH AutoFlow CO2 Air-Jacketed Incubator (NuAire, Plymouth, MN, USA). 0.01 g of graphene (pG, GO or rGO) powder was dissolved in 10 mL of milli-Q water to obtain concentration of 1000 ppm. Then, the prepared stocks of graphene were added to Dulbecco’s Modified Eagle’s culture Medium to obtain the concentration of 25 ppm of each type of graphene. U-87 MG glioblastoma cells were seeded in 75 cm^2^ culture flasks (1 × 10^6^ cells per flask) and incubated to reach 70% confluency. Then, the cells were washed by phosphate-buffered saline (Sigma-Aldrich) and subsequently Modified Eagle’s culture Medium with addition of graphene was added into each flask. After 24 h, cells were disassociated by 0.25% trypsin with EDTA, harvested and suspended in fresh DMEM medium. Then the cells were centrifuged at 1200 rpm for 5 min, washed with phosphate buffered saline and centrifuged to obtain a pellet. The cells were suspended in RNA later (Thermo Fisher Scientific, USA) and stored at 4 °C for the analysis. For the control, the cells were cultured in the Dulbecco’s modified Eagle’s culture medium containing 10% fetal bovine serum (Life Technologies, Houston, TX, USA) without the addition of graphene.

### RNA isolation

Total RNA was isolated from 1 × 10^6^ cells using Blood/Cell RNA Mini Kit (Syngen, Wroclaw, Poland) according to the manufacturer’s instructions. To remove potential traces of genomic DNA, the extracted RNA samples were treated with a TURBO DNA-*free*™ Kit (Ambion, Austin, TX, USA) for 30 min at 37 °C. RNA concentration was quantified by UV absorption (Nanodrop, LabTech International, UK) and RNA quality was assessed using an Agilent 2100 Bioanalyzer© and RNA 6000 Nano Kit (Agilent, Santa Clara, CA, USA). For all RNA samples, the RIN (RNA integrity number) value was above 8, ensuring the high quality of isolated RNA.

### Microarray analysis

Gene expression profiles were determined using GeneChip^®^ Human Gene 1.0 ST arrays (Affymetrix, Santa Clara, CA, USA) according to the manufacturer’s protocols. Briefly, 100 ng of total RNA per sample was reverse-transcribed, amplified, fragmented, and labeled using the Ambion^®^ WT Expression Kit and Affymetrix GeneChip^®^ WT Terminal Labeling Kit with included quality controls. Hybridization to the microarrays was conducted for 17 h at 45 °C in an Affymetrix GeneChip Hybridization Oven 640. Following hybridization, the microarrays were washed and stained on an Affymetrix GeneChip Fluidics Station 450 and scanned on an Affymetrix GCS 3000 GeneArray Scanner.

Quality analyses were performed using the Affymetrix Expression Console™ Software and standard Affymetrix quality metrics. Raw microarray data were background corrected, log transformed, and quantile normalized using the robust multi-array average (RMA) algorithm as implemented in the Partek^®^ Genomics Suite™ software (Partek Inc., St. Louis, MO, USA). The statistical significance of the results was estimated by analysis of variance (ANOVA). Differentially expressed genes were identified using the fold change (FC) of gene expression ratios > 1.5 and *P* value< 0.05 corrected for false discovery rate (FDR) as significance criteria.

### Validation of mRNA levels by RT-qPCR

To validate the microarray results, the expression levels of 10 selected genes in the cultured cells treated with GO (compared with cultured cells treated with buffer) were quantified by real-time reverse transcription-polymerase chain reaction (RT-qPCR). cDNA was synthesized from 400 ng of total RNA using the QuantiTect Reverse Transcription kit (Qiagen, Hilden, Germany) according to the manufacturer’s protocol. Amplification primers were designed using the Clone Manager Suite (Sci-Ed Software, Morrisville, USA). RT-qPCR assays were carried out using the LightCycler^®^480 and LightCycler^®^480 FastStart SYBR Green I Master (Roche Diagnostics GmbH, Germany). All assays were run in triplicate. Quantification cycles (Cq) were calculated using the *second derivative* method (LightCycler^®^480 Software, Roche). The fold change in gene expression levels, corrected by efficiency, was analyzed using Relative Expression Software Tool (REST 2009) (Qiagen; [57]). The expression data were normalized to the polymerase (RNA) II (DNA directed) polypeptide A (POLR2A) and ribosomal protein L29 (RPL29) genes, which in a RefFinder algorithm-based selection were the most stable among the 4 candidate reference genes tested. All experiments were performed according to the MIQE guidelines [58].

### Enrichment analysis

The list containing names of genes, the expression of which was regulated in GBM cells by GO treatment, was annotated with overrepresented (enriched) biological terms using the Enrichr tool (http://amp.pharm.mssm.edu/Enrichr/) [26]. Such terms include: (1) ontologies, which are defined groups of biological entities, such as “biological processes” or “molecular functions”; (2) transcriptional regulators, which are molecules crucial for modulating gene expression; (3) biochemical pathways; and (4) hub proteins, which interact with large numbers of molecular partners. Enrichr calculates *P*-values of enrichment using Fisher’s Exact Test. Only terms showing the statistical significance of enrichment of at least *P *= 0.05, after adjustment for multiple testing (Benjamini–Hochberg method), were considered to be genuinely enriched and were included in the results. The enrichment method for analysis of transcriptomic data is resistant to potential false positive findings, inevitably resulting from large scale microarray studies.

## Additional files

**Additional file 1: Table S1.** All probes reporting statistically significant difference between GBM cells treated with GO, GN or GR and non-treated GBM cells.

**Additional file 2: Table 2A.** All terms that were identified in enrichment analysis of the list of differentially expressed genes responding to GO. **Table 2B.** Non-redundant transcriptional regulators which target genes are overrepresented in the list of differentially expressed genes responding to GO.

## Abbreviations

GBM: glioblastoma multiforme
RT-qPCR: real-time reverse transcription polymerase chain reaction
OXPHOS: mitochondrial oxidative phosphorylation
GO: graphene oxide
rGO: reduced graphene oxide
pG: pristine grapheme
ROS: reactive oxygen species
CSC: cancer stem cells

## References

[1] Liu Z, Guo Z, Zhong H, Qin X, Wan M, Yang B (2013). Graphene oxide based surface-enhanced Raman scattering probes for cancer cell imaging. Phys Chem Chem Phys.

[2] Yang K, Feng L, Shi X, Liu Z (2013). Nano-graphene in biomedicine: theranostic applications. Chem Soc Rev.

[3] Yang K, Zhang S, Zhang G, Sun X, Lee ST, Liu Z (2010). Graphene in mice: ultrahigh in vivo tumor uptake and efficient photothermal therapy. Nano Lett.

[4] Akhavan O, Ghaderi E (2013). Graphene nanomesh promises extremely efficient in vivo photothermal therapy. Small.

[5] Akhavan O, Ghaderi E, Emamy H (2012). Nontoxic concentrations of PEGylated graphene nanoribbons for selective cancer cell imaging and photothermal therapy. J Mater Chem.

[6] Sun X, Liu Z, Welsher K, Robinson JT, Goodwin A, Zaric S, Dai H (2008). Nano-graphene oxide for cellular imaging and drug delivery. Nano Res.

[7] Akhavan O, Meidanchi A, Ghaderi E, Khoei S (2014). Zinc ferrite spinel-graphene in magneto-photothermal therapy of cancer. J Mater Chem.

[8] Geim AK, Novoselov KS (2007). The rise of graphene. Nat Mater.

[9] Jaworski S, Sawosz E, Grodzik M, Winnicka A, Prasek M, Wierzbicki M, Chwalibog A (2013). In vitro evaluation of the effects of graphene platelets on glioblastoma multiforme cells. Int J Nanomed.

[10] Mao HY, Laurent S, Chen W, Akhavan O, Imani M, Ashkarran AA, Mahmoudi M (2013). Graphene: promises, facts, opportunities, and challenges in nanomedicine. Chem Rev.

[11] Dreyer DR, Park S, Bielawski CW, Ruoff RS (2010). The chemistry of graphene oxide. Chem Soc Rev.

[12] Sanchez VC, Jachak A, Hurt RH, Kane AB (2012). Biological interactions of graphene-family nanomaterials: an interdisciplinary review. Chem Res Toxicol.

[13] Hinzmann M, Jaworski S, Kutwin M, Jagiello J, Kozinski R, Wierzbicki M, Grodzik M, Lipinska L, Sawosz E, Chwalibog A (2014). Nanoparticles containing allotropes of carbon have genotoxic effects on glioblastoma multiforme cells. Int J Nanomed.

[14] Sawosz E, Jaworski S, Kutwin M, Hotowy A, Wierzbicki M, Grodzik M, Kurantowicz N, Strojny B, Lipinska L, Chwalibog A (2014). Toxicity of pristine graphene in experiments in a chicken embryo model. Int J Nanomed.

[15] Jaworski S, Sawosz E, Kutwin M, Wierzbicki M, Hinzmann M, Grodzik M, Winnicka A, Lipinska L, Wlodyga K, Chwalibog A (2015). In vitro and in vivo effects of graphene oxide and reduced graphene oxide on glioblastoma. Int J Nanomed.

[16] Szmidt M, Sawosz E, Urbanska K, Jaworski S, Kutwin M, Hotowy A, Wierzbicki M, Grodzik M, Lipinska L, Chwalibog A (2016). Toxicity of different forms of graphene in a chicken embryo model. Environ Sci Pollut Res Int.

[17] Chatterjee N, Eom HJ, Choi J (2014). A systems toxicology approach to the surface functionality control of graphene-cell interactions. Biomaterials.

[18] Akhavan O, Ghaderi E, Akhavan A (2012). Size-dependent genotoxicity of graphene nanoplatelets in human stem cells. Biomaterials.

[19] Jarosz A, Skoda M, Dudek I, Szukiewicz D (2016). Oxidative stress and mitochondrial activation as the main mechanisms underlying graphene toxicity against human cancer cells. Oxid Med Cell longev.

[20] Zhou H, Zhang B, Zheng J, Yu M, Zhou T, Zhao K, Jia Y, Gao X, Chen C, Wei T (2014). The inhibition of migration and invasion of cancer cells by graphene via the impairment of mitochondrial respiration. Biomaterials.

[21] Zhou T, Zhang B, Wei P, Du Y, Zhou H, Yu M, Yan L, Zhang W, Nie G, Chen C (2014). Energy metabolism analysis reveals the mechanism of inhibition of breast cancer cell metastasis by PEG-modified graphene oxide nanosheets. Biomaterials.

[22] Chen GY, Chen CL, Tuan HY, Yuan PX, Li KC, Yang HJ, Hu YC (2014). Graphene oxide triggers toll-like receptors/autophagy responses in vitro and inhibits tumor growth in vivo. Adv Healthcare Mater.

[23] Grodzik M, Sawosz E, Wierzbicki M, Orlowski P, Hotowy A, Niemiec T, Szmidt M, Mitura K, Chwalibog A (2011). Nanoparticles of carbon allotropes inhibit glioblastoma multiforme angiogenesis in ovo. Int J Nanomed.

[24] Szmidt M, Urbańska K, Grodzik M, Orłowski P, Sawosz E, Wierzbicki M, Sysa P (2012). Morphology of human glioblastoma model cultured in ovo. Bull Vet Inst Pul.

[25] Fiorillo M, Verre AF, Iliut M, Peiris-Pages M, Ozsvari B, Gandara R, Cappello AR, Sotgia F, Vijayaraghavan A, Lisanti MP (2015). Graphene oxide selectively targets cancer stem cells, across multiple tumor types: implications for non-toxic cancer treatment, via “differentiation-based nano-therapy”. Oncotarget.

[26] Chen EY, Tan CM, Kou Y, Duan Q, Wang Z, Meirelles GV, Clark NR, Ma’ayan A (2013). Enrichr: interactive and collaborative HTML5 gene list enrichment analysis tool. BMC Bioinform.

[27] Liu L, Ryu S, Tomasik MR, Stolyarova E, Jung N, Hybertsen MS, Steigerwald ML, Brus LE, Flynn GW (2008). Graphene oxidation: thickness-dependent etching and strong chemical doping. Nano Lett.

[28] Akhavan O, Ghaderi E, Aghayee S, Fereydoonia Y, Talebia A (2012). The use of a glucose-reduced graphene oxide suspension for photothermal cancer therapy. J Mater Chem.

[29] Yan T, Skaftnesmo KO, Leiss L, Sleire L, Wang J, Li X, Enger PO (2011). Neuronal markers are expressed in human gliomas and NSE knockdown sensitizes glioblastoma cells to radiotherapy and temozolomide. BMC Cancer.

[30] Allen M, Bjerke M, Edlund H, Nelander S, Westermark B (2016). Origin of the U87MG glioma cell line: good news and bad news. Sci Transl Med.

[31] Shen H, Liu M, He H, Zhang L, Huang J, Chong Y, Dai J, Zhang Z (2012). PEGylated graphene oxide-mediated protein delivery for cell function regulation. ACS Appl Mater Interfaces.

[32] Choi YJ, Kim E, Han J, Kim JH, Gurunathan S (2016). A novel biomolecule-mediated reduction of graphene oxide: a multifunctional anti-cancer agent. Molecules.

[33] Mallineni SS, Shannahan J, Raghavendra AJ, Rao AM, Brown JM, Podila R (2016). Biomolecular interactions and biological responses of emerging two-dimensional materials and aromatic amino acid complexes. ACS Appl Mater Interfaces.

[34] Votyakova TV, Reynolds IJ (2001). DeltaPsi(m)-Dependent and -independent production of reactive oxygen species by rat brain mitochondria. J Neurochem.

[35] Breuer ME, Willems PH, Smeitink JA, Koopman WJ, Nooteboom M (2013). Cellular and animal models for mitochondrial complex I deficiency: a focus on the NDUFS4 subunit. IUBMB Life.

[36] Pramanik KC, Boreddy SR, Srivastava SK (2011). Role of mitochondrial electron transport chain complexes in capsaicin mediated oxidative stress leading to apoptosis in pancreatic cancer cells. PLoS ONE.

[37] Bhattacharya K, Bag AK, Tripathi R, Samanta SK, Pal BC, Shaha C, Mandal C (2014). Mahanine, a novel mitochondrial complex-III inhibitor induces G0/G1 arrest through redox alteration-mediated DNA damage response and regresses glioblastoma multiforme. Am J Cancer Res.

[38] Lloyd RE, Keatley K, Littlewood DT, Meunier B, Holt WV, An Q, Higgins SC, Polyzoidis S, Stephenson KF, Ashkan K (2015). Identification and functional prediction of mitochondrial complex III and IV mutations associated with glioblastoma. Neuro-oncology.

[39] He J, Yang J, Chen W, Wu H, Yuan Z, Wang K, Li G, Sun J, Yu L (2015). Molecular features of triple negative breast cancer: microarray evidence and further integrated analysis. PLoS ONE.

[40] Xu H, Ma J, Wu J, Chen L, Sun F, Qu C, Zheng D, Xu S (2016). Gene expression profiling analysis of lung adenocarcinoma. Braz J Med Biol Res.

[41] Griguer CE, Cantor AB, Fathallah-Shaykh HM, Gillespie GY, Gordon AS, Markert JM, Radovanovic I, Clement-Schatlo V, Shannon CN, Oliva CR (2013). Prognostic relevance of cytochrome C oxidase in primary glioblastoma multiforme. PLoS ONE.

[42] Suzuki C, Daigo Y, Kikuchi T, Katagiri T, Nakamura Y (2003). Identification of COX17 as a therapeutic target for non-small cell lung cancer. Can Res.

[43] Huang G, Ho B, Conroy J, Liu S, Qiang H, Golubovskaya V (2014). The microarray gene profiling analysis of glioblastoma cancer cells reveals genes affected by FAK inhibitor Y15 and combination of Y15 and temozolomide. Anticancer Agents Med Chem.

[44] Hurtado-Lopez LM, Fernandez-Ramirez F, Martinez-Penafiel E, Carrillo Ruiz JD, Herrera Gonzalez NE (2015). Molecular analysis by gene expression of mitochondrial ATPase subunits in papillary thyroid cancer: Is ATP5E transcript a possible early tumor marker?. Med Sci Monit Int Med J Exp Clin Res.

[45] Hanahan D, Weinberg RA (2011). Hallmarks of cancer: the next generation. Cell.

[46] Chen Z, Lu W, Garcia-Prieto C, Huang P (2007). The Warburg effect and its cancer therapeutic implications. J Bioenerg Biomembr.

[47] Son MJ, Woolard K, Nam DH, Lee J, Fine HA (2009). SSEA-1 is an enrichment marker for tumor-initiating cells in human glioblastoma. Cell Stem Cell.

[48] Janiszewska M, Suva ML, Riggi N, Houtkooper RH, Auwerx J, Clement-Schatlo V, Radovanovic I, Rheinbay E, Provero P, Stamenkovic I (2012). Imp2 controls oxidative phosphorylation and is crucial for preserving glioblastoma cancer stem cells. Genes Dev.

[49] Vlashi E, Lagadec C, Vergnes L, Matsutani T, Masui K, Poulou M, Popescu R, Della Donna L, Evers P, Dekmezian C (2011). Metabolic state of glioma stem cells and nontumorigenic cells. Proc Natl Acad Sci USA.

[50] Yamaguchi H, Condeelis J (2007). Regulation of the actin cytoskeleton in cancer cell migration and invasion. Biochem Biophys Acta.

[51] Seabra AB, Paula AJ, de Lima R, Alves OL, Duran N (2014). Nanotoxicity of graphene and graphene oxide. Chem Res Toxicol.

[52] Lammel T, Boisseaux P, Fernandez-Cruz ML, Navas JM (2013). Internalization and cytotoxicity of graphene oxide and carboxyl graphene nanoplatelets in the human hepatocellular carcinoma cell line Hep G2. Part Fibre Toxicol.

[53] Li Y, Liu Y, Fu Y, Wei T, Le Guyader L, Gao G, Liu RS, Chang YZ, Chen C (2012). The triggering of apoptosis in macrophages by pristine graphene through the MAPK and TGF-beta signaling pathways. Biomaterials.

[54] Zhao J, Zhang J, Yu M, Xie Y, Huang Y, Wolff DW, Abel PW, Tu Y (2013). Mitochondrial dynamics regulates migration and invasion of breast cancer cells. Oncogene.

[55] Boland ML, Chourasia AH, Macleod KF (2013). Mitochondrial dysfunction in cancer. Front Oncol.

[56] Veatch JR, McMurray MA, Nelson ZW, Gottschling DE (2009). Mitochondrial dysfunction leads to nuclear genome instability via an iron-sulfur cluster defect. Cell.

[57] Pfaffl MW, Horgan GW, Dempfle L (2002). Relative expression software tool (REST) for group-wise comparison and statistical analysis of relative expression results in real-time PCR. Nucleic Acids Res.

[58] Bustin SA, Benes V, Garson JA, Hellemans J, Huggett J, Kubista M, Mueller R, Nolan T, Pfaffl MW, Shipley GL (2009). The MIQE guidelines: minimum information for publication of quantitative real-time PCR experiments. Clin Chem.

